# Association between fundus sex index and hypertension, diabetes mellitus, and smoking in the Kumejima study

**DOI:** 10.1007/s10384-026-01334-3

**Published:** 2026-03-04

**Authors:** Takehiro Yamashita, Ryo Asaoka, Aiko Iwase, Hiroshi Sakai, Hiroto Terasaki, Taiji Sakamoto, Makoto Araie

**Affiliations:** 1 Department of Ophthalmology, Kagoshima University Graduate School of Medical and Dental Sciences, Kagoshima, Japan; 2 Department of Ophthalmology, Seirei Hamamatsu General Hospital, Shizuoka, Japan; 3 Tajimi Iwase Eye Clinic, Gifu, Japan; 4 Urasoe Sakai Eye Clinic, Okinawa, Japan; 5 Department of Ophthalmology, Kanto Central Hospital, Tokyo, Japan

**Keywords:** Fundus sex index, Fundus photographs, Smoking, Hypertension, Diabetes

## Abstract

**Purpose:**

Fundus parameters can be used to quantify masculinity or femininity as a fundus sex index (FSI) ranging from 0 to 1. The purpose of this study was to investigate the association between smoking, hypertension, diabetes, and FSI in the Kumejima population study

**Study design:**

Prospective cross-sectional observational population study

**Methods:**

Using color fundus photographs obtained from the Kumejima population study, 1653 healthy right eyes with reliable fundus parameter measurements were included. The tessellation fundus index R/(R + G + B), red-green-blue intensity in eight locations around the optic disc and foveal region, optic disc ovality ratio, papillomacular angle, and retinal vessel angles were quantified using ImageJ. The FSI was calculated using machine learning from the 42 fundus parameters, and the Mann–Whitney U-test was used to assess whether there were differences in the FSI depending on the presence or absence of smoking, hypertension, and diabetes

**Results:**

The mean age of the 838 men and 815 women included in this study was 52.8 and 54.0 years, respectively. The FSI of the smoking group was lower than of the non-smoking group overall (P < 0.001). The FSI of the hypertension and diabetes groups was significantly higher than of the non-hypertension (P = 0.005) and non-diabetes (P = 0.017) groups only in women

**Conclusion:**

In the Kumejima population study of healthy eyes in individuals aged 40 years or older, the fundus tended to be more feminine in women with hypertension or diabetes than in those without these conditions

## Introduction

Analyses of the UK Biobank and EyePACS datasets show that deep-learning artificial intelligence (AI) can accurately predict several demographic and systemic features. For instance, it estimates age with a mean absolute error (MAE) of 3.26 years and determines sex with an area under the curve (AUC) of 0.97. Furthermore, the model achieved a mean absolute error of 11.23 mmHg in systolic blood pressure prediction and yielded AUC values of 0.71 and 0.70 for identifying smoking status and cardiac complications, respectively, by analyzing color fundus photographs using the Inception-v3 model [1]. Furthermore, refraction can be estimated by AI through the TensorFlow platform with an MAE of 0.56 diopters, as demonstrated using the UK Biobank and Age-Related Eye Disease Study datasets [2]. In addition, analysis of the Beijing Eye Study revealed that the Inception-ResNet-v2 architecture can predict axial length directly from fundus photographs, achieving an MAE of 0.56 mm [3].

Sex prediction by AI can reach an accuracy of up to 97% [1]. Nevertheless, determining the specific features responsible for this prediction remain elusive, even when attention heat maps or quantitative analyses are applied. A similar limitation is observed in board games such as chess, Go, and Shogi: deep-learning systems can consistently suggest optimal moves, yet the rationale behind these decisions is often unclear [4]. Because of this opacity, experts must analyze AI’s strategies, models frequently referred to as “black-box AI” [5]. To shed light on this black box, classical statistical approaches such as multivariable regression have been considered as explanatory alternatives to AI [5]. The ocular fundus, much like a fingerprint or facial features, is unique to each individual. Several characteristics show wide variation: the angle and path of retinal vessels [6–10], the location and morphology of the optic disc [11, 12], and the coloration of the peripapillary area [13–16]. In particular, the trajectories of the arcade vessels parallel those of the infra- and supra-temporal retinal nerve fiber layers, and both shift toward the fovea as axial length increases [6–10]. Our previous studies demonstrate that L2-regularized ridge regression based on fundus parameters could predict sex with accuracies of 77.9% in young adults in their 20s [17], 63.2% in children with a mean age of 8.5 years [18], and 80.4% in individuals older than 40 years [19]. Because a multivariable regression approach was employed, sex-related differences in ocular features were also identified: women exhibited more oval-shaped optic discs, retinal vessels positioned closer to the fovea, and a greener peripapillary fundus color compared with men [17–19]. We also revealed age-specific and axial length-specific fundus changes using this method [20, 21].

In a previous study, regression analysis yielded a predictive score (0–1) for each fundus image: lower values indicated a more masculine fundus, while higher values reflected a more feminine fundus. This approach treats sex as a continuous parameter based on fundus characteristics, rather than a binary classification. Thus, the probability score can be used to evaluate sex-related tendencies rather than categorical differences. The prediction score was designated as the fundus sex index (FSI), and its correlation with sex-specific axial length was assessed in the Kumejima population. Fundi with more masculine features were consistently linked to greater axial length across both sexes [22].

However, the broader meaning of the FSI remains unclear, and further research is needed on gender-specific behavior and diseases. One behavior pattern that shows gender differences is smoking, which is more prevalent among men [23]. Additionally, diseases such as hypertension and diabetes, although dependent on age, are more prevalent among men [24–27]. In this study, we investigated the association between smoking, hypertension, diabetes, and FSI in healthy eyes of individuals aged 40 years or older in the Kumejima population study.

## Methods

### Study population

The Kumejima Study conformed to the tenets of the Declaration of Helsinki and regional regulations; the ethics committee of the local government & health department (Kumejima Town) approved the study protocol. Informed written consent was obtained from all participants prior to examination. The study was performed between May 2005 and August 2006. All Kumejima residents aged 40 years or older were invited to participate after being informed of the study protocol. According to the official household registry, 5249 residents met this age criterion in 2005. Of these, 617 individuals could not be examined for specific reasons, leaving 4632 eligible participants [28, 29].

Participants were excluded if they had insufficient fundus images; pseudophakic or aphakic eyes; spherical equivalent refractive error less than –8 D or greater than +5 D; optic nerve, retinal, or brain disease; unclear peripheral fundus areas; or unmeasurable fundus parameters [19].

### Examinations and diagnosis

Screening consisted of a structured interview, as described in detail elsewhere [28, 29]. Ophthalmologists and trained examiners performed a full ocular evaluation, including best corrected visual acuity, refractive error, intraocular pressure (IOP), central corneal thickness, anterior chamber depth, and axial length. Slit-lamp biomicroscopy, gonioscopy, ophthalmoscopy, fundus photography, and perimetry were also performed.

Stereoscopic 30° and 45° color fundus photographs were obtained with a non-mydriatic fundus camera (ImageNet TRC-NW7; Topcon). Refraction was assessed using an auto-refractometer (ARK-730, Topcon), and IOP was measured with a Goldmann applanation tonometer, using the median of repeated measures. Central corneal thickness was measured by specular microscopy (SP-2000, Topcon), and anterior chamber depth and axial length with an IOLMaster (Carl Zeiss Meditec). The van Herick method was applied for peripheral anterior chamber depth, while Shaffer’s grading with a Goldmann two-mirror lens was used for gonioscopy.

Visual field testing employed FDT perimetry with the C-20-1 screening program. Whenever ocular abnormalities were suspected, a more detailed examination was arranged, including slit-lamp biomicroscopy, gonioscopy, fundus assessment, and Humphrey Field Analyzer Central 24-2 SITA Standard testing. The methods for optic disc, fundus, and visual field evaluation, as well as glaucoma diagnosis, have been published elsewhere [28, 29]. The diagnosis of hypertension was based on systolic pressure ≥140 mmHg or diastolic pressure ≥90 mmHg [25], or the use of blood pressure–lowering medication. Smoking status and the presence of diabetes were assessed by interview.

### Measurement of fundus parameters

In total, 42 fundus parameters were evaluated, as described previously (Fig. 3) [19]. The angles of the supratemporal (ST) and infratemporal (IT) major retinal arteries (RA) and veins (VA) relative to the temporal horizontal line were determined (ST-RA, IT-RA, ST-VA, IT-VA) [6, 7, 10]. The papillomacular position (PMP) was defined as the angle between the horizontal axis and a line drawn from the optic disc center to the fovea [11]. Disc ovality was expressed as the ratio of the minimum to maximum disc diameter [12]. Mean red, green, and blue intensities were calculated for the fovea and eight peripapillary sectors. The tessellation fundus index (TFI) was calculated at each of the nine regions using the formula: TFI = R/(R + G + B), where R, G, and B represent the mean intensities of the red, green, and blue channels, respectively [13, 14].

Supratemporal and infratemporal retinal artery angles (ST-RA and IT-RA) were identified with red double arrows, and retinal vein angles (ST-RV and IT-RV) with blue double arrows. The PMP was marked by a white double arrow. The ovality ratio of the optic disc was obtained by dividing the shortest diameter by the longest. Mean RGB intensities and TFI values were obtained for the fovea and eight peripapillary regions. Eight circles, each 96 pixels in diameter, were placed around the optic disc, with the lateral circle centered on the axis from the fovea to the optic nerve head. A 32-pixel circle was additionally positioned at the fovea, as attention maps from deep-learning models indicated this region’s significance for sex identification [1].

Color fundus images were analyzed using ImageJ software (version 1.47, National Institutes of Health, Bethesda, MD, USA; http://imagej.nih.gov/ij/). Semi-automated extraction of the 42 color fundus parameters was performed using the macro function, after the fovea, optic nerve head edge, and the intersections of a 208-pixel circle centered on the optic disc with the superolateral and inferolateral vessels were identified.

### Statistical analyses

As reported previously, sex classification was performed using L2-regularized binomial logistic (ridge) regression with 42 input variables, including RGB intensities and TFI values at nine sites, ST-RA, IT-RA, ST-VA, IT-VA, PMP, and ovality ratio [19]. This approach applies an L2 penalty to regression coefficients, thereby reducing the risk of overfitting often encountered in conventional multivariate or logistic regression analyses. The penalty term constrains the sum of squared coefficients to stabilize estimation [30, 31]. Specifically, the penalized log-likelihood function was maximized using the formula below:

$$\sum_{i=1}^{n}[(yixi \beta -\mathrm{log}(1+{e}^{xi\beta })]- \lambda \sum_{j=1}^{p}{\beta }_{j}^{2}$$

Here, xi denotes the i-th row of the matrix containing n observations and p predictors; β represents the column vector of regression coefficients; and λ is the penalty term. Leave-one-out cross-validation was applied to assess the diagnostic performance of ridge binomial logistic regression [19], with each eye serving once as the validation case (1653 iterations). In each iteration, the λ value was chosen as the penalty term yielding the smallest prediction error within the training dataset (1653 eyes). Model accuracy was quantified using the area under the receiver operating characteristic curve (AROC). The final model was trained using all 1653 eyes. The resulting predictive value, ranging from 0 to 1, was defined as the FSI, representing the degree of fundus masculinity or femininity [22]. The model and TFI values were identical to those used in references 19 and 22. Eyes with an FSI approaching 0 were characterized as having more masculine fundus features, whereas those with an FSI near 1 were considered more feminine. Differences between men and women in age and FSI were examined using the Mann–Whitney U-test, and differences between men and women in smoking, hypertension, and diabetes were assessed using the chi-square test. The Mann–Whitney U-test was also used to evaluate differences in FSI based on the presence or absence of smoking, hypertension, and diabetes. As the average age of menopause for Japanese women is 50 years [32], a similar analysis was performed after dividing women into those younger than 50 years and those 50 years or older. A p value <0.05 was considered statistically significant. All analyses were conducted using SPSS Statistics v19 for Windows (SPSS Inc., IBM) and R v3.1.3 (R Foundation for Statistical Computing). Ridge regression was performed using the ‘glmnet’ package. Leave-one-out cross-validation was conducted in R without additional packages.

## Results

Among the 4632 eligible residents, 3762 individuals (81.2%) underwent examination. The photographs obtained from 376 right and 421 left eyes were excluded because of cataract, corneal opacity, large pterygium, or a small pupil. Eyes with prior intraocular surgery (453 right, 443 left) were also excluded. Furthermore, eyes were excluded when the refractive error was greater than −8 or +5 diopters (14 right eyes, 11 left eyes) or when optic disc diseases, including glaucoma, retinal disease, or brain disease, were present in either eye (711 right eyes, 679 left eyes). Only the right eyes were used to measure the fundus parameters. Of the 2208 normal eyes, 555 eyes were excluded because the fundus parameters could not be measured due to unclear peripheral fundus area or an unidentifiable fovea. Detailed inclusion and exclusion criteria, as well as definitions of acceptable study eyes, have been reported elsewhere [19–22]. Ultimately, 1653 right eyes (838 men and 815 women) were included in the analysis. Table 1 summarizes the demographic characteristics of the analyzed eyes. No significant age difference was observed between the sexes (p = 0.14), whereas the FSI was significantly greater in women than in men (p < 0.001). Men were more likely to smoke (p < 0.001) and had a higher prevalence of diabetes (p < 0.001). There was no significant difference between the sexes in the prevalence of hypertension (p = 0.13).

**Table 1:** Demographic information of the right eyes of the 1653 eligible participants

	Men	Women	*P* value
Number of eyes	838	815	
Age (years)	52.8 ± 9.4	54.0 ± 10.8	0.14
Fundus sex index	0.35 ± 0.23	0.64 ± 0.23	<0.001*
Smoking (yes/no (%))	582/256 (69.5%)	123/692 (15.1%)	<0.001*
Hypertension (yes/no (%))	263/575 (31.4%)	228/587 (28.0%)	0.13
Diabetes (yes/no (%))	69/769 (8.2%)	27/788 (3.3%)	<0.001*

* *P* < 0.05, Chi-square test or Mann–Whitney U-test

Differences in the fundus sex index between participants with and without smoking, hypertension, and diabetes are shown in Table 2. Overall, smokers showed a significantly reduced FSI relative to non-smokers (p < 0.001), but no significant difference was observed in the FSI between smokers and non-smokers when stratified by sex (men: p = 0.16; women: p = 0.67). There was no significant difference in the FSI between the presence or absence of hypertension in the overall group (p = 0.37) or in men only (p = 0.96). However, in women only, the FSI was significantly higher in the hypertension group than in the non-hypertension group (p = 0.005). There was no significant difference in the FSI between the presence or absence of diabetes in the overall group (p = 0.88) or in men only (p = 0.14). However, in women only, the FSI was significantly higher among individuals with diabetes than among non-diabetic participants (p = 0.017).

**Table 2:** Difference in fundus sex index between groups

		Yes	No	*P* value
Smoking	Overall	0.39 ± 0.26	0.57 ± 0.26	<0.001*
	Men	0.34 ± 0.23	0.36 ± 0.23	0.16
	Women	0.65 ± 0.22	0.64 ± 0.23	0.67
Hypertension	Overall	0.50 ± 0.28	0.49 ± 0.27	0.37
	Men	0.35 ± 0.23	0.35 ± 0.23	0.96
	Women	0.68 ± 0.22	0.63 ± 0.23	0.005*
Diabetes	Overall	0.50 ± 0.29	0.49 ± 0.27	0.88
	Men	0.40 ± 0.27	0.34 ± 0.23	0.14
	Women	0.74 ± 0.19	0.64 ± 0.23	0.017*

* *P* < 0.05, Mann–Whitney U-test

Differences in the fundus sex index between participants with and without hypertension or diabetes among women aged in their 40s and those aged 50 years or older are shown in Table 3. Among women in their 40s, there was no significant difference in the FSI between those with hypertension (p = 0.82) or diabetes (p = 0.89) compared with those without these conditions. However, in women aged 50 years or older, the FSI was significantly higher in those with hypertension (p < 0.001) or diabetes (p = 0.008) compared to those without.

**Table 3:** Difference in fundus sex index between groups in women aged 40 years and over 50 years

		Yes	No	*P* value
Hypertension	40s	0.64 ± 0.23 (43 eyes)	0.64 ± 0.23 (313 eyes)	0.82
	50 or older	0.69 ± 0.21 (185 eyes)	0.62 ± 0.23 (274 eyes)	<0.001*
Diabetes	40s	0.64 ± 0.25 (6 eyes)	0.64 ± 0.23 (350 eyes)	0.89
	50 or older	0.77 ± 0.16 (21 eyes)	0.64 ± 0.23 (438 eyes)	0.008*

* *P* < 0.05, Mann–Whitney U-test

## Discussion

To the best of our knowledge, this is the first report to investigate the relationship between the presence or absence of smoking, hypertension, and diabetes and the FSI. Overall, the FSI of the smoking group was significantly more masculine than of the non-smoking group, but no significant difference was observed within each sex. These results suggest that although there is a sex difference in smoking rates, there is no tendency for individuals with a more masculine fundus to be more likely to smoke.

No significant difference in FSI was found between participants with and without hypertension or diabetes in the overall group or among men. Among women, however, fundus appearance tended to be more feminine in those with hypertension or diabetes than in those without these conditions. Female hormones reportedly have protective effects against hypertension and diabetes [33–36]. Women who undergo menopause at age 40 or younger, whether surgically or naturally, and postmenopausal women have an increased risk of hypertension and diabetes [33–35], whereas hormone therapy reduces this risk in postmenopausal women [33, 36]. Although no studies have directly examined the relationship between the FSI and female hormones, findings from our previous cohort study show that the accuracy of sex determination using fundus parameters increased with age, reaching 56.3% at 8.5 years and 73.1% at 11.5 years. Because secondary sexual characteristics typically emerge around age 10 [37], these differences in fundus appearance may be influenced by sex hormones. Therefore, if a more feminine fundus reflects higher levels of female hormones, our finding in women was contrary to expectations.

The Kumejima Study is a population-based study targeting people aged 40 years or older. Because female hormone levels decline rapidly with menopause [32, 33], we divided women at the average menopausal age of 50 years [32] and analyzed them separately. Among women in their 40s, there was no significant difference in FSI between those with and without diabetes or hypertension. However, among women aged 50 years or older, those with hypertension or diabetes exhibited a significantly more feminine fundus than those without. If a more feminine FSI reflects higher female hormone levels, this result is plausible. In addition to female hormones, factors such as smoking, obesity, a sedentary lifestyle, and hyperlipidemia are involved in the development of hypertension and diabetes [33]. It is possible that women with high hormone levels prior to menopause, despite these other factors, did not develop hypertension or diabetes until after menopause, when hormonal protection diminished. Previous hypertension studies support this finding. Among individuals aged 40–49 years, the prevalence of hypertension in women is about 10% lower than in men, whereas in the 70–74 age group, the prevalence is almost identical between sexes [24]. However, these interpretations remain hypothetical, and the relationship between the FSI and female hormones needs to be investigated further.

Sex has traditionally been analyzed as a nominal variable. However, the FSI allows the degree of masculinity or femininity in fundus appearance to be evaluated as a continuous variable, even within a group of only men or only women. In diseases with known sex differences, such as macular holes, which are more common in women, both men and women with a more feminine fundus may be more susceptible. Examining the association between FSI and macular holes separately by sex could reveal new fundus characteristics related to disease development. Additionally, in behaviors with sex differences, such as career selection, men who choose female-dominated professions, such as nursing, may have a more feminine fundus. Given that women with masculine characteristics and men with feminine characteristics certainly exist, the FSI may serve as a new research tool in medicine and behavioral science.

This study has some limitations. First, the accuracy of the FSI is limited. While AI models can determine sex from fundus photographs with 97% accuracy [1], multiple regression analysis achieved only 80.4% accuracy [19], suggesting that current parameters may not fully capture sex-related differences. Second, the assessment of smoking, hypertension, and diabetes relied on self-reported interviews, introducing the possibility of recall bias or misclassification. This concern is particularly relevant for diabetes, which was not confirmed by blood tests. Furthermore, the number of cases with diabetes was small, with only 69 men and 27 women, reducing statistical reliability. Multiple testing also increases the risk of chance findings, particularly in subgroup analyses with small sample sizes. In a multiple regression analysis using FSI as the dependent variable and sex, age, smoking, hypertension, diabetes, sex × hypertension, and sex × diabetes as independent variables, only sex significantly correlated with FSI. Therefore, confirmatory studies that include more diseased eyes are needed. Third, this study included only healthy eyes and excluded eyes with retinal or optic nerve disease, it remains unknown whether FSI can be accurately measured in diseased eyes or whether systemic disease influences FSI differently in such cases. Fourth, FSI is a new indicator, and research to date has been limited to the Kumejima study. External validity must be evaluated using additional databases, as cohort-specific characteristics (older age, ethnic homogeneity) may contribute to model overfitting. Findings should therefore be considered exploratory until independently validated.

In conclusion, we investigated the association between smoking, hypertension, diabetes, and FSI in the Kumejima population study of individuals aged 40 years or older. The fundus tended to be more feminine in women with hypertension or diabetes, particularly those aged 50 or older, than in those without these conditions. This method may be useful for future research on sex-related tendencies in various diseases and behaviors.
